# Right Information, Right Patient, Right Time: Utilizing the MyCareCompass Platform to Deliver Patient Education in the Oncology Setting

**DOI:** 10.1007/s13187-023-02350-4

**Published:** 2023-08-04

**Authors:** Linda Fleisher, Cassidy Kenny, Cheryl Rusten, Daniella Koren, Zoe Landau

**Affiliations:** 1 Fox Chase Cancer Center, Cancer Prevention and Control, PA Philadelphia, USA; 2 ARCHES Health Technology, NY New York, USA

**Keywords:** Cancer Education, Digital Health, Patient Anxiety

## Abstract

Patient education is an important part of cancer care as it increases patient activation and informed decision-making, reduces anxiety, and improves outcomes. However, challenges to providing appropriate education to patients exist on both the health provider and patient side of the equation, e.g., time constraints and health literacy issues. Digital health education is a fast-growing field of interest that has been shown to improve health outcomes, increase effectiveness of medical treatments and education, lower medical costs, and enhance both clinical diagnosis and research opportunities by streamlining data collection, sharing, and analysis. In 2019, Fox Chase Cancer Center was selected by ARCHES, an established patient education software company, to pilot its award-winning digital patient engagement system MyCareCompass. During the pilot, patients scheduled for port insertions were sent electronic messages inviting them to review various online educational materials related to their procedure and subsequent concerns. The invitations and resources were seamlessly integrated into the scheduling system and timed to arrive when patients would most need them. There was high usage of the port-related materials and patients reported a high level of satisfaction with the delivery system and the information. This automated process of delivering high-quality and relevant patient education was able to be implemented smoothly with IT involvement, had a positive impact on patients without adding any extra burden to the care team, and highlighted opportunities to integrate these types of interventions into routine care.

## Introduction

Patient education is vital to optimal cancer care as it drives patient activation and informed decision-making, reduces anxiety, and improves outcomes [1]. Research has shown that patients who are well-informed about their cancer and treatment are more likely to follow their treatment regimen and have positive outcomes after cancer and are also less likely to go to the emergency room when experiencing symptoms [2–4]. Digital health is a rapidly expanding field of interest in the patient education sphere that has been shown to improve health outcomes, increase effectiveness of medical treatments and education, lower medical costs, and enhance research opportunities by streamlining data collection, sharing, and analysis, as well as clinical diagnosis and decision-making [4, 5]. Digital intervention has been found to positively impact patients’ quality of life, satisfaction, and lifestyle across diagnoses and socioeconomic status [6]. It allows for more streamlined connection between patients and providers, especially but not only in the face of a disaster such as the COVID-19 pandemic, by increasing access to help with self-management and health behavior change [7–9]. With the growing number of people across socioeconomic strata utilizing smart phones, including those from underserved populations, digital health can allow for convenient, effective, and accessible healthcare to the majority of the world’s population [10, 11].

Delivery of appropriate cancer education can be a challenge on several levels. Health care professionals are often racing the clock and may not have the time to fully educate patients about their condition or treatments [12]. In addition, they may not have access to credible relevant content, especially since such content is constantly being updated as new information is learned [13]. Even when they do have the time and the content, there is a tendency to overload patients with all the information at once, rather than parceling it out across the continuum of care, which tends to cause information avoidance, confusion, and noncompliance with treatment or health behavior recommendations among patients [14, 15]. Other barriers on the patient side can include limited health literacy, lack of time, information overload, distractions, cultural differences, fatigue, and anxiety [12, 16]. To be most effective, the ideal model for patient education would be “the right information, to the right patient, at the right time” [17].

Digital health education continues to emerge as an approach that can address these provider and patient challenges and includes both web-based interventions and mobile/online health tools. The aim of digital health tools is to supplement learning outcomes and promote informed decision-making in patients through shared information, knowledge, and support [18]. As technology capabilities at health systems continue to become stronger and more powerful, digital education is becoming more accessible as these new systems allow for more interactive ways of educating patients and tailoring to their individual needs [19]. Done well, digital health education allows patients to consume the education at the right time, in the right place, and at their own pace, without adding extra burden to the clinical care team [8]. It allows patients to have the information provided as needed, rather than overwhelming them with too much at once. They can also return to the materials at their convenience and can share them with their families and caregivers. This is especially important as cancer therapies continue to shift towards home care, and the entire at-home care team needs to understand the cancer treatment and monitoring needs. Our recent experience with the COVID pandemic and lack of face-to-face access to health care providers provides just one good example of how digital education can be valuable [7, 8].

Fox Chase Cancer Center (Fox Chase), one of the National Cancer Institute’s Comprehensive Cancer Centers, has long valued and implemented evidence-based cancer education to patients through a variety of channels, including within the clinic setting. The Resource and Education Center (REC), established in 2000, is one example of this commitment to patient education. The REC is located within the clinic setting as well as online and provides patients with vetted information about diagnoses, treatment, and self-care [20]. In addition, our population science researchers have developed and tested numerous digital health tools to support cancer patients and continue to explore innovative methods and approach to inform patients about their cancer diagnosis and treatment [21–23].

In 2019, ARCHES (an established patient education software company) was awarded the C3Prize from Astellas Pharma Inc. for Non-Rx Oncology Innovation [24, 25], and Fox Chase was selected as a pilot site to launch their digital patient engagement system called MyCareCompass (MCC). ARCHES’ software platform is HIPAA compliant, integrates with all EHR systems in the market, and is designed to increase patient satisfaction, improve clinical workflow, boost treatment adherence, and empower patients throughout their cancer journey. The platform uses videos, emails, and text messaging to deliver personalized education to patients and caregivers at the exact time they need it, based on select real-time EHR data points. It offers multiple educational modules for patients undergoing infusion chemotherapy, immunotherapy, port-insertion, and surgical ostomies. ARCHES curated this content based on oncology industry insights and over a decades-worth of normative data. MCC also offers some psycho-social resources that ARCHES created in collaboration with Cancer Care, a national organization providing free, professional support services and information to help people manage the emotional, practical, and financial challenges of cancer [26].

The overall purpose of the pilot was to assess the feasibility of integrating this software with Fox Chase’s electronic health record system and to evaluate patient usage and perception of value of the platform in order to provide insights for broader implementation of additional patient education modules through this platform in the future. After reviewing MCC’s vast library of content (video, text, and graphics delivered via both text and email messages), the Fox Chase multi-disciplinary stakeholder team decided to implement the port insertion module for the pilot. Patients are often unaware and/or fearful of this procedure and education about it would be quite helpful to prepare patients and lower their anxiety [1]. In addition, this group of patients could be easily identified in the EHR scheduling system, hopefully ensuring a less complicated integration process. In addition to the port-related videos and materials, the team selected additional MCC modules (seeking social support, nutritional, and financial resources) to provide additional supplemental resources to patients during their treatment. Optional surveys to solicit user feedback were embedded in the delivery system.

## Aim/Purpose

This pilot (December 2020 through the end of 2022) had three key aims as follows:

1. To determine the feasibility of implementing and integrating the MCC platform into Fox Chase’s existing electronic health record system
2. To determine how patients would actually utilize the educational material provided to them
3. To determine patients’ perceptions of the value of the educational material and their satisfaction with the education delivery process

## Methods

### Planning for Implementation

**Fig. 1:**
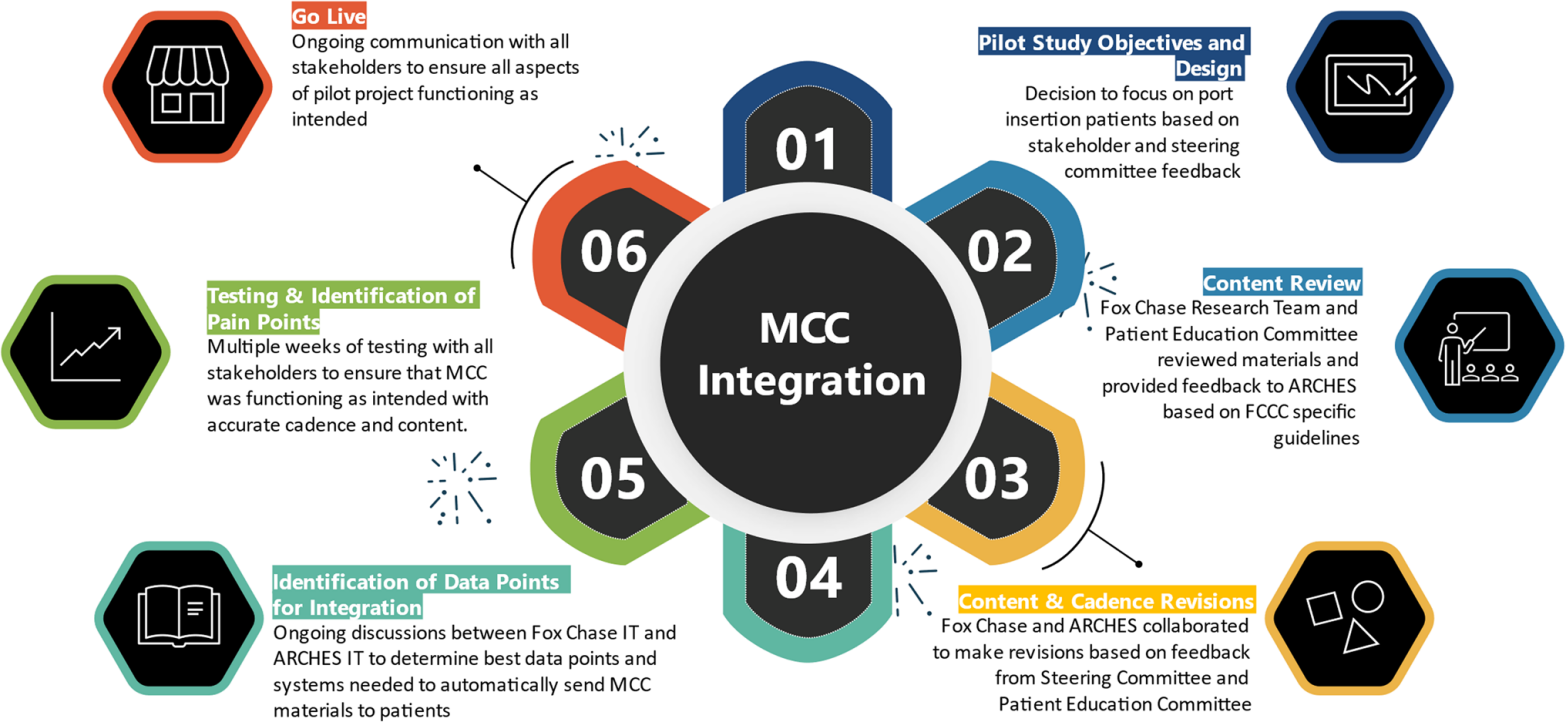
MCC integration process

At project inception, a multidisciplinary steering committee (Committee) comprised of Fox Chase’s and ARCHES’ members was established to guide the project. Fox Chase members included representatives from essential relevant clinical and non-clinical areas including nursing, IT, patient education, and population science. Representatives from ARCHES included a project manager, a technical lead, and the company founder. As shown in Fig. 1, the integration of a robust platform such as MCC requires multiple planning steps with a multi-disciplinary team from both the health care organization and the digital health company.

### Content Review

A key early decision by the Committee was to focus on just one educational module, supplemented by several psycho-social resources. This would simplify the integration process and allow for more focused usage and evaluation review. As noted, the Committee selected the port insertion module because there was clinical and patient education buy-in, and the platform could be clearly connected to Fox Chase’s electronic scheduling system. At the time, this system was separate from the full EMR. Three psycho-social modules were also selected: two existing MCC modules on nutrition and finances, respectively, and a module on resources that was modified to include specific Fox Chase resources, such as the social work department. The Committee, along with the research team, reviewed this material and made changes to assure alignment with internal guidelines and health literacy requirements.

### Description of MCC Modules and Surveys

The port insertion module consists of 3 videos delivered via email and SMS directly to patients scheduled for the procedure. The first is a “welcome” video that explains what material will be forthcoming and when and gives patients the opportunity to opt-out of receiving any additional information. The second is a pre-insertion video called *What to Expect with Your Port Insertion*. This video explains what a port is, why it might be needed, how it works, and how it is inserted. The final video, entitled *What to Expect After Your Port Insertion,* explains how to take care of the port site.

The first psycho-social material offered was a curated list of various other Fox Chase resources available to patients (e.g., social work services and transportation assistance). The second focused on good nutrition care during treatment, and the third was about finding financial support. These psycho-social materials were in a PDF format.

Short surveys already embedded in the MCC platform followed the port insertion education and the psycho-social modules. Working collaboratively with ARCHES, the Committee made minor revisions to these surveys. Additionally, the Fox Chase team wanted to gather further qualitative insights from patients who used the MCC platform. To accomplish this, they developed an email/text message to be sent through the MCC platform that invited patients interested in participating in a short telephone survey to send their contact information via a link to a REDCap contact form. Those who provided their contact information were called by the research team and asked several open-ended questions in a short telephone interview to gather additional insights. Demographic data were collected with these patients including age, identified gender, identified race and ethnicity, and a health literacy screener.

### Cadence

**Fig. 2:**
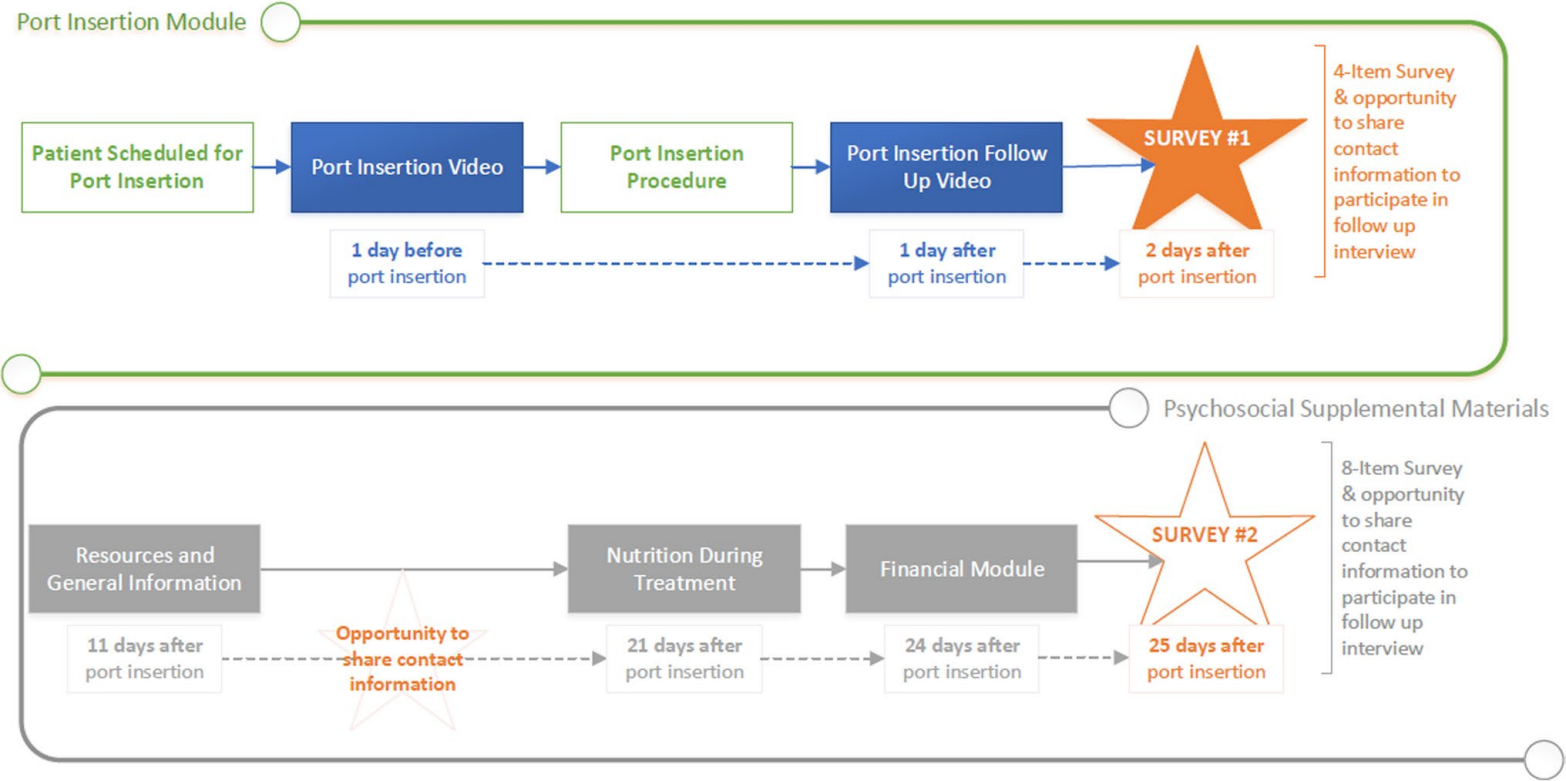
Cadence

As shown in Fig. 2, we developed the cadence for delivering the modules and feedback surveys to meet the current clinical scheduling and workflow.

Patients received the initial welcome video when they were scheduled for a port insertion, the pre-insertion video the day before the appointment, and the post-insertion video a day after the insertion. Two days later, they received a 4-question survey assessing the value of the program including specific questions about the videos. Those survey results were collected directly by ARCHES’ software platform and were available to Fox Chase via a data dashboard updated in real time. Along with the survey, patients received their invitation to participate in a follow-up telephone survey and to share contact information with the research team if interested. The additional psycho-social materials and final survey were sent over a period of 25 days following review of the port insertion education.

### Data Points for Integration

As the content development and revision work progressed, Fox Chase’s and ARCHES’ teams worked in tandem to identify the data exchange process to automatically send the MCC educational material to patients and to integrate the invitation to participate in the qualitative study into the rollout. Delivery methods were to include both email and SMS options. This process entailed identifying the data points and systems that would deliver the information to the right patients at the right time and also provide needed information to the research team for the qualitative study. It included technology teams in both organizations to work to identify specific data from the scheduling system to select appropriate patients and technology approaches for secure data sharing between the health system and the MCC platform. Over a period of 6 months, these processes were developed and tested with the various stakeholders to assure everything was working properly.

## Results

### Integration Feasibility

We were able to successfully set up and integrate the data exchange without issue, in large part, due to the buy-in of all stakeholders and particularly the Fox Chase IT team. It also was dependent on the careful planning and testing of the process and systems prior to launch.

### Usage

Usage data for both the port insertion and psycho-social modules was collected between February 1, 2021, and December 30, 2022. Over 1200 patients were scheduled for port insertions and received the MCC materials. Less than 1% of these patients unsubscribed from the program. As shown in Table 1, most patients had both an email and mobile telephone number in the EHR system and received the messages in both forms.

**Table 1:** Message delivery mediums

Patients scheduled for port insertion	1272
Unsubscribed	11 (<1%)
Received only texts	355 (28%)
Received both texts and emails	917 (72%)

**Fig. 3:**
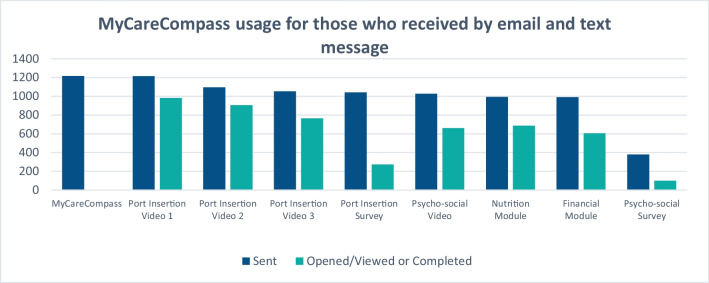
MCC usage

As can be seen in Fig. 3 below, patients were very engaged in the port-related messages and materials. There was less usage of the additional psycho-social modules, but over half of the patients who received these modules did view the information. Opened/viewed rates were not captured on the initial introduction of the tool - only the subsequent videos, surveys, and modules.

### Patient Impact and Value

Survey data were collected from two sources, the platform-embedded surveys and the qualitative telephone interview. As shown in Table 2, the survey was voluntary and almost 20% of those who received the port modules completed the related surveys. Of those, 72% gave the highest rating to the statement that the MCC information helped to ease their fear and anxiety about the procedure. About 4 out 5 respondents gave the highest rating to the value of MCC in preparing for and understanding the procedure and understanding how to care for the port.

**Table 2:** Results from the Port Insertion Survey

Received port insertion materials	*N* = 1272 (%)
Clicked “Take the survey”	315 (24%)
Completed surveys	235 (18.5%)
Port survey responses	N = 235 (%)
MCC provided value in helping to ease my fear and anxiety about the port insertion procedure	
Highly dissatisfied	3 (1.3%)
Somewhat dissatisfied	3 (1.3%)
Neither satisfied nor dissatisfied	12 (5.1%)
Somewhat satisfied	46 (19.6%)
Highly satisfied	167 (71.1%)
Missing	4 (1.7%)
The port insertion video helped me get prepared for my procedure	
Highly dissatisfied	4 (1.7%)
Somewhat dissatisfied	5 (2.1%)
Neither satisfied nor dissatisfied	12 (5.1%)
Somewhat satisfied	35 (14.9%)
Highly satisfied	179 (76.2%)
The MCC emails and videos helped me understand why I need a port	
Highly dissatisfied	2 (<1%)
Somewhat dissatisfied	6 (2.6%)
Neither satisfied nor dissatisfied	17 (7.2%)
Somewhat satisfied	33 (14%)
Highly satisfied	177 (75%)
The port insertion video helped me to better understand how to care for my port	
Highly dissatisfied	4 (1.7%)
Somewhat dissatisfied	8 (3.4%)
Neither satisfied nor dissatisfied	14 (6%)
Somewhat satisfied	39 (16.6%)
Highly satisfied	170 (72.3%)

As shown in Table 3, the response rate for the psycho-social modules was much lower with a less than 10% response rate. Of those who responded, 60% strongly agreed that the MCC information had a positive impact on them and helped them find ways to cope with their cancer. We asked what other type of information would they be interested in and managing side effects and nutrition had the highest rating.

**Table 3:** Results from the Psychosocial Modules Survey

Psychosocial survey response rates	N = 1272 (%)
Clicked “Take the survey”	167 (13.1%)
Completed surveys	87 (6.8%)
Psycho social survey responses	N = 87 (%)
Overall, the MCC program had a positive impact on my experience with cancer	
Strongly disagree	4 (4.6%)
Somewhat disagree	3 (3.4%)
Do not agree or disagree	9 (10.3%)
Somewhat agree	16 (18.4%)
Strongly agree	52 (59.8%)
Missing	3 (3.4%)
The MyCareCompass communications helped me find ways to cope with cancer	
Strongly disagree	2 (2.3%)
Somewhat disagree	0
Do not agree or disagree	9 (10.3%)
Somewhat agree	20 (22.3%)
Strongly agree	56 (64.4%)
What other topics would you be interested in receiving information on? Check all that apply	
Clinical trials	23 (26.4%)
Immunotherapy	29 (33.3%)
Managing treatment side effects	65 (74.7%)
Nutrition during treatment	54 (62.1%)
Rehabilitation services	17(19.5%)

### Follow-up Interview Results

The follow-up interview data were collected between February 2021 and June 2022 with the goal of obtaining deeper insights about the MCC platform and content. A total of 20 patients opted to participate in this survey, which represents less than 10% of potential eligible participants who completed the feedback surveys. The responses were recorded, transcribed, and analyzed to identify themes.

All the patients who were interviewed remembered watching the port insertion video, while only 18% remembered the emailed information on support and nutrition. The majority (79%) stated the port videos made them feel less anxious and more prepared. They shared:

- “I didn’t feel anxious after I saw it. It was very straightforward and made me feel ok about having it done.”
- “[It made me] more comfortable. I think I was a little more prepared. I wasn’t as scared.”
- “I thought it was very helpful because I knew what I was up against, and it all went well.”
- “I really liked the video because what I had in my head was not what happened”

Very few patients stated it initially increased their anxiety.

Consistent with the survey findings, patients valued receiving the information in real time and the MCC platform was well received.

- “I thought the whole thing was excellent. Very impressed with how quickly I received the first one. I found it all very timely. I thought they were very good- don’t think I would have changed anything.”
- “I liked that it was convenient and it was very user friendly. If I wanted to watch the video it was easy to just click on the link. There was nothing I didn’t like”
- “The information beforehand was very helpful and helped me feel prepared, the information I had beforehand and stories I heard was not what actually happened - the videos portrayed exactly what happened”

In response to our request for suggestions, patients shared:

- “It would be helpful if the oncologist told the patient they were going to be receiving additional support.”
- “I think more could been given about after care. I had a lot of questions which were answered, but how to care for it after would have been helpful as well.”
- “I think the videos could be a bit more engaging - they are very simplistic.”

The overwhelming majority (94%) thought the emails/texts were easy to use, and 100% would recommend MCC to a friend if they were getting ready for a procedure.

## Discussion

Integrating a robust digital health platform into our cancer center’s patient care workflow was quite successful from the standpoint of technology as well as clinical and patient acceptability. We found that it was feasible to implement and integrate the MCC platform into the cancer center’s electronic medical system without major issues or problems. However, this implementation depended on a dedicated IT champion who worked closely with various IT teams to design the data exchange and test the system. This IT champion actually became a key member of the multi-disciplinary planning committee that guided the project. We believe that assembling a multi-disciplinary team that included key leadership from nursing, IT, and population research was critical to establishing buy-in and providing the decision-making authority to move the project forward, and ultimately, the platform was well received by the entire team for a number of reasons. The content was based on health communications’ best practices such as utilization of multi-media, incorporation of evidence based content, and attention to health literacy needs. The platform was designed to be adaptable and could be modified for specific work flows and content based on Fox Chase’s requirements. ARCHES’ credibility bolstered by its receipt of a competitive innovation award that funded the pilot project, and its willingness to collaborate with our team was foundational. The learning from the implementation supports key factors in program implementation that can be applied to other digital health tools. This includes involving champions from various sectors of the organization in all phases of the project, the reputation and collaborative style of the third-party vendor, technology that is designed for integration into work flows, and evidence-based and engaging content. We found that multi-disciplinary champions working collaboratively ensured that the integration was seamless and that patients could be delivered “the right information at the right time.”

The MCC platform and modules were also well received and valued by our patients. Not only did we find that very few patients unsubscribed to the emails and text messages, but the majority that opened the port insertion materials also told us that they were highly satisfied with them and overall found the material reduced their anxiety about what to expect. This tells us that patients want and will use digital educational information if it is relevant to their needs and provided at the “right time.” The population of patients who received the messages was all patients who were scheduled for a port insertion and included a diverse patient population, negating some beliefs that certain groups will not use digital avenues. The usage data for the port insertion material was exceptionally high in comparison to the subsequent modules such as the psycho-social material, confirming that the timeliness and relevancy of the information is critical to patient engagement. It could also reflect a preference to view video material as opposed to PDF documents, which these were. Moreover, of those who participated in the surveys, over 90% reported that the port insertion content eased their fears about the procedure and helped them to be prepared. Qualitative data from the follow-up interviews were consistent in supporting the value of MCC. Of those patients who completed the survey post the psychosocial module, the majority reported that they strongly agreed that the MCC materials had a positive impact on their experience with cancer and helped them find ways to cope.

This platform and other well designed digital health tools for patients have the potential to address health equity among underserved communities who may have less access to vetted and evidence-based patient education. Smart phones and other technologies have become ubiquitous among all populations groups and are essential to our everyday lives [11]. Receiving critical health information from a trusted source directly to your phone or email with no additional log-ins or advertisements could be a game changer in reaching those less often reached through other methods.

## Next Steps

Overall, this project was so successful that, even though the pilot is over, Fox Chase is continuing to use the modules and platform surveys as part of standardized care for all patients scheduled for a port insertion. As we expand the initiative, we launched a second pilot with urology ostomy modules in April 2023 and are exploring additional opportunities to leverage the extensive content library within the platform. Currently, we have conducted a series of meetings with additional departments across the health system that have expressed interest in MyCareCompass and see the value of leveraging the platform for a variety of patient education and engagement efforts. As we move to scaling the use of the platform and designing workflows, content and patient journeys to specific patient needs, we have identified senior leadership champions to facilitate the implementation, including financing, processes, and priorities for broader implementation. Our goal is to utilize the MyCareCompass platform across a broad array of oncology clinical programs as well as to plan future research and program evaluation focusing on differences in uptake and satisfaction among subgroups of patients across gender, race/ethnicity, disease type, and other psychosocial factors.
