# Correction to: Right Information, Right Patient, Right Time: Utilizing the MyCareCompass Platform to Deliver Patient Education in the Oncology Setting

**DOI:** 10.1007/s13187-023-02371-z

**Published:** 2023-09-14

**Authors:** Linda Fleisher, Cassidy Kenny, Cheryl Rusten, Daniella Koren, Zoe Landau

**Affiliations:** 1 Fox Chase Cancer Center, Cancer Prevention and Control, Philadelphia, PA USA; 2 ARCHES Health Technology, New York, NY USA

**Correction to: Journal of Cancer Education**

**https://doi.org/10.1007/s13187-023-02350-4**

The original version of this article unfortunately contained a mistake.

The acknowledgment should read: We would like to acknowledge Stephanie Raivitch, Director of Fox Chase Cancer Center’s Resource Education Center and the Patient Education Committee and Michael Nichols for their ongoing support in piloting the MyCareCompass platform.

The original article has been corrected.

